# Economic Inequality Increases Status Anxiety Through Perceived Contextual Competitiveness

**DOI:** 10.3389/fpsyg.2021.637365

**Published:** 2021-05-24

**Authors:** Davide Melita, Guillermo B. Willis, Rosa Rodríguez-Bailón

**Affiliations:** Department of Social Psychology, Faculty of Psychology, Mind, Brain and Behavior Research Center (CIMCYC), University of Granada, Granada, Spain

**Keywords:** status anxiety, economic inequality, socioeconomic status, competitiveness, wellbeing

## Abstract

Status anxiety, the constant concern about individuals’ position on the social ladder, negatively affects social cohesion, health, and wellbeing (e.g., chronic stress). Given previous findings showing that status anxiety is associated with economic inequality, we aimed in this research to test this association experimentally. A cross-sectional study (Study 1) was run in order to discard confounding effects of the relationship between perceived economic inequality (PEI) and status anxiety, and to explore the mediating role of a competitive climate (*N* = 297). Then we predicted that people assigned to a condition of high inequality would perceive more status anxiety in their social context, and they would themselves report higher status anxiety. Thus, in an experimental study (Study 2) PEI was manipulated (*N* = 200). In Study 1, PEI uniquely predicted status anxiety, and perceived competitiveness mediated the relationship. In Study 2 PEI increased perceived contextual status anxiety, a specific form of perceived competitiveness based on socioeconomic status (SES). Moreover, preliminary evidence of an indirect effect was found from PEI to personal status anxiety, through (higher) perceived contextual status anxiety. These preliminary findings provide experimental evidence for the effects of economic inequality on status anxiety and the mechanism involved. Economic inequality makes people feel that they live in a society where they are constantly concerned and competing with each other for their SES. These results could have important implications as health and wellbeing could be promoted by reducing economic inequalities and the competitive and materialistic environments of our societies.

## Introduction

Most modern societies are living in the most unequal time since the industrial revolution (Piketty, 2013). Economic inequality has been related with political polarization (Winkler, 2019), impaired democracy (Krieger and Meierrieks, 2016), and poorer health and wellbeing (Layte, 2012). All in all, it has been suggested that economic inequality increases social dysfunction (Pickett and Wilkinson, 2015).

Although many of these consequences are derived from the accumulation of power by an elite group of individuals acting on behalf of their own interests (Stiglitz, 2012), it has been argued that psychological processes must be taken into account in order to explain some of the negative effects of economic inequality (e.g., over mental health; Layte, 2012). One of these processes, according to the Spirit Level approach (Wilkinson and Pickett, 2017; see also Buttrick et al., 2017), may be status anxiety (SA)—the tendency to worry constantly about one’s own socioeconomic position and about socioeconomic success according to social standards (De Botton, 2004).

Status anxiety has been associated with income inequality across a variety of cross-sectional studies (Layte and Whelan, 2014; Delhey et al., 2017; Melita et al., 2020), but up to now, a causal relationship has not yet been demonstrated. The main goal of the present research is to provide experimental evidence about the causal effect of economic inequality on SA.

## Economic Inequality and Status Anxiety

Social context shapes norms about which social categories are more relevant to make sense of the social world (Fiske and Neuberg, 1990); in turn, some social categories may become more chronically accessible and central to social identity (Oakes, 1987). Independent of their socioeconomic status (SES), people living in unequal countries tend to be more sensitive to hierarchies and status cues (e.g., Kraus et al., 2017), and to be more stressed when perceiving high inequality (Wilkinson and Pickett, 2009; Sprong et al., 2019). As such, it is plausible that higher perceived economic inequality (PEI) will lead people to attribute more importance to their SES and to worry more about the position they occupy on the social ladder (De Botton, 2004).

Building on this idea, the SA theory posits that when economic distances are higher, SES—that is, one’s status based on the economic dimension—gains a more relevant role in our perception of self-worth and wellbeing relative to other values and parameters (Walasek and Brown, 2019). Thus, when economic inequality is higher, people are more chronically concerned about their SES. According to SA theory, a person who lives in a society with large income disparities, for instance, would probably feel a considerable pressure to achieve an equal or better salary than similar others. Moreover, as SES is a relative attribute that expresses one’s rank in a given society or reference group, when inequality increases, so does the tendency to social comparison. More than absolute economic resources, relative economic position is what determines our life satisfaction (Cheung and Lucas, 2016).

In fact, preliminary evidence supports these notions. For instance, in more unequal countries, there is greater interest in status-signaling goods, and people spend more money on the lottery; all this may indicate a greater importance of the social position and economic success (Bol et al., 2014; Walasek and Brown, 2015). In experimental settings, it has been found that participants bet more money and assume more risks when they perceive higher inequality in a gambling game (Payne et al., 2017). These effects may also appear in other risk-taking behaviors such as crime, acquisition of debt, and unhealthy behaviors (e.g., drug consumption) because people strain to obtain greater reward in order to achieve perceived social standards of socioeconomic success (Payne, 2017).

Cross-sectional studies have directly examined the relationship between income inequality and SA. Among European citizens, for instance, regardless of their SES, those who live in more unequal countries report a higher degree of feeling that other people look down on them because of their job or income, and are found to report higher status seeking, both being considered as expressions of higher SA (Paskov et al., 2013; Layte and Whelan, 2014). Moreover, SA could cause harmful chronic stress reactions (Marmot, 2004) and unadaptive coping strategies (e.g., risk-taking behaviors). In fact, in large cross-country observational studies, SA mediated the negative effects of inequality on well-being (Delhey and Dragolov, 2014) and depression (Layte, 2012). However, when including variation over time within countries, observational studies found opposite results, indicating that European citizens living in more unequal countries feel less motivated to improve their SES, as it seems to become an unreachable goal for most (Paskov et al., 2017).

Importantly, being immersed in an economic context perceived as highly unequal can shape descriptive norms about how people in that context tend to relate to each other. For instance, PEI has been found to increase the belief that the normative climate is individualistic and competitive, generating a highly demanding social environment that could lead to more competitive behaviors (Sommet et al., 2019; Sánchez-Rodríguez et al., 2019). Furthermore, social comparison, although distinct from competitiveness, is an important source of competitive behavior (García et al., 2013). Thus, given that SES becomes a relevant dimension of comparison as inequality increases, people may feel that they are competing with each other in order to maintain or increase their SES, and they may feel more pressure to obtain or borrow more resources than others do, and signal a higher material standing. Hence, we maintain that PEI could increase the perception of a social environment in which people are concerned about their SES and compete with each other for a better position (i.e., a social context where others are perceived as having higher SA). Ultimately, we hypothesized that PEI increases both personal SA and perceived contextual SA. As in previous studies (e.g., Layte and Whelan, 2014), we expect these effects to happen along the entire social ladder.

Similarly, as the social context could exhort a great influence on attitudes and motivational orientation (Cialdini et al., 1991; Sommet et al., 2019), perceiving similar others to be highly concerned about their SES could lead to an SES-competitive mindset that further boosts personal SA. In the present research, we explored the role of perceived contextual SA as mediator in the effect of PEI on personal SA.

## The Present Research

In this paper we present an exploratory cross-sectional and a preregistered experimental study to examine whether PEI influences SA. Moreover, we investigated whether PEI increases perceived contextual SA (as indicated by similar others’ perceived SA), and whether this variable could mediate the aforementioned effect between PEI and SA.

Given that PEI has been demonstrated to affect the way people perceive their social world and how they interact within it, we adapted a consolidated experimental paradigm to manipulate it (Sánchez-Rodríguez et al., 2019). All presented protocols and studies were approved by the ethical committee of the authors’ university of affiliation.

## Study 1

We ran an exploratory cross-sectional study in order to test the role of PEI in the prediction of SA. Although the relationship between PEI and SA has already been established in previous studies (Melita et al., 2020), we aimed to exclude possible confounding effects, and explore the predictive validity of PEI on SA, controlling for perceived competitive climate and for other variables that are theoretically related to PEI and SA, namely, SES and political orientation^1^.

### Method

#### Participants

This study was part of a larger set of studies. Participants were recruited in a bus station in a city in the South of Spain, and those who were working for an organization that had at least three other employees were assigned to another study (focused on organizational settings), whereas participants who did not meet this criterion were assigned to the current study.

After granting informed consent, 309 participants completed a short paper-pencil questionnaire (*M*_*estimated time*_ = 10 min). We excluded 12 cases for not answering one of the focal variables, or failing to answer an attention check item correctly. The final sample was composed of 297 participants, 108 students, 79 unemployed, 110 employed, self-employed or retired, 56% women (*M*_*age*_ = 29.86; SD_*age*_ = 13.21). Participants in each quintile of household income from the bottom to the top were 30, 18, 8, 12, and 11%, respectively (21% did not indicate their household income).

#### Measures

*Status anxiety:* Participants completed the Spanish Version of the Status Anxiety scale (Keshabyan and Day, 2020; Melita et al., 2020). The scale consists of five items and participants were instructed to rate their agreement with each item on the scale from 1 (*totally disagree*) to 7 (*totally agree*). Examples of items included the following: “I worry that my social status will not change”, and “I sometimes worry that I might become lower in social standing” (α = 0.86; *M* = 4.04, SD = 1.69).

*Perceived economic inequality* was indicated by averaging PEI in Spain in general and in a set of reference groups to which people usually compare themselves (i.e., their friends, family, schoolmates, and neighbors; Alderson and Katz-Gerro, 2016). Combining both the local and societal levels of PEI increases the generalizability of results (García-Castro et al., 2019). The items were adapted from a question used in the International Social Survey Program and in studies about PEI (e.g., Castillo et al., 2012; Shariff et al., 2016). Participants answered to what extent they agreed with the following assertions: “Differences in income <in Spain/among people in the reference group> are too large”, from 1 (*totally disagree*) to 7 (*totally agree*). The resulting five items loaded on a single factor in EFA, explaining 46% of the variance (α = 0.70; *M* = 4.34, SD = 1.08).

*Perceived competitive climate* was indicated by averaging perceived competitiveness in Spain and in the same reference groups to which PEI was also asked. Participants answered to what extent they agreed with the following sentences: “I feel that <in Spain/among people in the reference group>, we are competing with each other”, from 1 (*totally disagree*) to 7 (*totally agree*). The resulting five items loaded on a single factor in EFA, explaining 45% of the variance (α = 0.70; *M* = 3.40, SD = 1.11).

*Political orientation* was measured by a single item asking participants to place themselves on a scale from 1 (*far left*) to 7 (*far right*; *M* = 3.41, SD = 1.45).

*Participants’ SES* was indicated by their educational attainment (indicated on a scale from 1, ‘‘*primary education*’’, to 8, ‘‘*doctoral degree*’’) and their household disposable income decile, which referred to Spanish income distribution^2^.

*Subjective SES* was measured using the MacArthur scale (Adler et al., 2000): a single item asking participants to place themselves according to their socioeconomic standing on a ladder with 10 steps representing society. (1 indicated those at the bottom, and 10 indicated those at the top; *M* = 5.48, SD = 1.54).

Finally, participants indicated their age, sex and work status. All materials and data are available at https://osf.io/h35uj/?view_only=c026d785644948ea945650cb88aa5ff3.

### Analyses

We ran a least squares linear regression analyses on SA in R (R Core Team, 2020). Then, we performed a bootstrap regression analysis in Macro Process for SPSS (Hayes, 2017) to examine whether PEI had an indirect effect on SA through a perceived competitive climate.

Multiple imputation by chained equations (MICE) was used to account for missing values of seven control variables, which ranged from 4% (sex) to 21% (household disposable income). The conclusions were the same regardless MICE and the control variables.

### Results

As shown in Table 1, both PEI and perceived competitive climate significantly and uniquely predicted SA scores in Model 2, that is, participants with higher scores in either of the two variables reported higher SA, independent of their sex, age, political orientation and SES.

**TABLE 1:** Regression analyses’ results using status anxiety as the criterion.

	**Model 1**		**Model 2**	
**Predictor**	**b**	**b 95% CI [LL, UL]**	**b**	**b 95% CI [LL, UL]**
(Intercept)	4.41**	[2.99, 5.83]	1.75*	[0.15, 3.35]
Female	0.48*	[0.09, 0.86]	0.37*	[0.00, 0.73]
Age	0.00	[−0.01, 0.02]	0.00	[−0.02, 0.02]
Students^a^	0.37	[−0.21, 0.95]	0.22	[−0.34, 0.78]
Unemployed^a^	0.43	[−0.09, 0.95]	0.39	[−0.11, 0.90]
Political orientation	0.03	[−0.10, 0.17]	0.07	[−0.06, 0.20]
Education	−0.16*	[−0.30, −0.02]	−0.13*	[−0.26, −0.00]
Income decile	–0.05	[−0.12, 0.02]	–0.04	[−0.11, 0.02]
Subjective SES	–0.03	[−0.17, 0.12]	–0.00	[−0.14, 0.13]
Perceived economic inequality			0.26**	[0.07, 0.44]
Perceived competitive climate			0.39**	[0.20, 0.57]
Fit	*R*^2^ = 0.090**		*R*^2^ = 0.207**	
	95% CI [0.02, 0.13]		95% CI [0.11, 0.26]	
Difference			Δ*R*^2^ = 0.117**	
			95% CI [0.05, 0.18]	

A significant *b*-weight indicates the semi-partial correlation is also significant. *b* represents unstandardized regression weights. *Indicates *p* < 0.05. ** indicates *p* < 0.01. ^*a*^Contrasted against employed, self-employed or retired.

**FIGURE 1:**
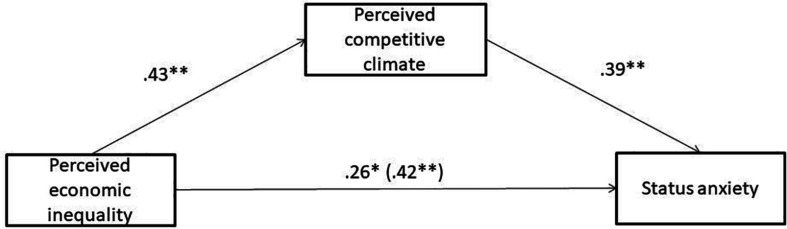
Indirect effect of perceived economic inequality on status anxiety through perceived competitive climate in study 1. Coefficients are standardized; total effect in parenthesis; ^∗^*p* < 0.01; ^∗∗^*p* < 0.001.

In addition, perceived competitive climate partially mediated the effect of PEI on SA (completely standardized indirect effect = 0.11; 95%CI [0.05, 0.17]; RMSEA = 0.063; CFI = 0.860; TLI = 0.835; SRMR = 0.064; Figure 1), as PEI predicted a higher perceived competitive climate, and this in turn predicted higher SA.

## Study 2

In this study, PEI was manipulated using an adaptation of the Bimboola Paradigm (Jetten et al., 2015; Sánchez-Rodríguez et al., 2019). The aim of the study was to provide experimental evidence about the effects of economic inequality on both perceived others’ and participants’ own SA^3^.

### Preregistered Hypotheses

We predicted that participants assigned to the high (vs. Low) inequality condition would report higher SA (H1), and would attribute more SA to other people who belong to their own income group (H2).

### Method

#### Participants

Given that we performed multiple hypotheses testing (i.e., two), we preregistered and applied a Bonferroni correction by setting hypotheses two-tailed testing α value at 0.025 (Bland and Altman, 1995). With this alpha, we calculated with G^∗^power (RRID:SCR_013726; Faul et al., 2007) that the sample size required for the 80% statistical power to detect a medium effect size (*d* = 0.50) would be *N* = 156. To that end, data collection would run until we reached a minimum of 156 valid observations and a maximum of 200.

The experiment was administered to 244 Spanish undergraduate students, aged between 18 and 30. Thirty-two cases were excluded from the final sample following pre-registration because they failed to answer the attention check correctly, and 12 participants were excluded from the final sample because they already took part in other similar studies, involving manipulations of PEI. The final sample consisted of 200 participants (*M*_*age*_ = 21.59; SD_*age*_ = 2.45; 44.5% women). With this final sample size, and α = 0.025, we were able to detect a minimum effect size (*d*) as big as 0.44 with 80% power.

#### Procedure

Participants completed an online survey and were randomly assigned to one of the two experimental conditions: low or high inequality. In both conditions, participants were instructed to imagine they would be starting a new life in a fictitious society called Bimboola, and they were asked to choose some goods from a list in order to start their new lives (a house, a car and a holiday trip). We informed them that this society was divided into three income groups, and we emphasized that people from each income group could choose only from a subset of goods (e.g., whereas the richest group could choose any type of house, including the best ones, the poorest group could choose only between the cheapest houses). The only differences between the low and high inequality conditions were the monthly earnings of the lowest and highest income groups and the type of goods they can afford.

Importantly, participants’ SES and their perceived mobility in Bimboola were kept constant across conditions by assigning participants to the same middle income group (i.e., Group 2), with the same amount of monthly income, and highlighting that in Bimboola there is a high chance to climb to an upper—or to descend to a lower—income group, according to one’s effort and work. All instructions about Bimboola were reinforced with infographics (instructions and infographics are available at OSF^4^).

#### Measures

*Participants’ expected status anxiety:* Participants completed the same SA scale as in Study 1, thinking about how they would feel in Bimboola (α = 0.85).

*Perceived contextual status anxiety* was indicated by perceived SA among members of participants’ income group in Bimboola (i.e., Group 2). To that end, participants were instructed to rate their agreement with the items of an adapted version of the same SA scale as in Study 1, thinking about how other people belonging to their same income group would feel in Bimboola (α = 0.82).

*Manipulation check:* PEI in Bimboola was measured by two items asking to what extent participants perceived the presented society as equal (reversed)/unequal (ranging from 1, “little equal/unequal”, to 9, “highly equal/unequal”). Items were highly correlated (*r* = 0.92), so we averaged them.

*Participants’ SES* was indicated by their household disposable income decile, referred to Spanish income distribution (19% bottom quintile of income, 19% second quintile, 19% middle quintile, 11% fourth quintile, and 15% top quintile, 18% did not indicate their household income).

Other variables not relevant to our hypotheses were measured with exploratory purposes^4^.

### Results

#### Preregistered Analyses

##### Manipulation check

Participants in the low inequality condition perceived Bimboola as significantly less unequal (*M* = 4.07; SD = 1.95) than participants in the high inequality condition (*M* = 8.13; SD = 1.35), *t*(165.01) = 16.98, *p* < 0.001; *d* = 2.44; 95%CI [2.07, 2.80].

##### Status anxiety

**FIGURE 2:**
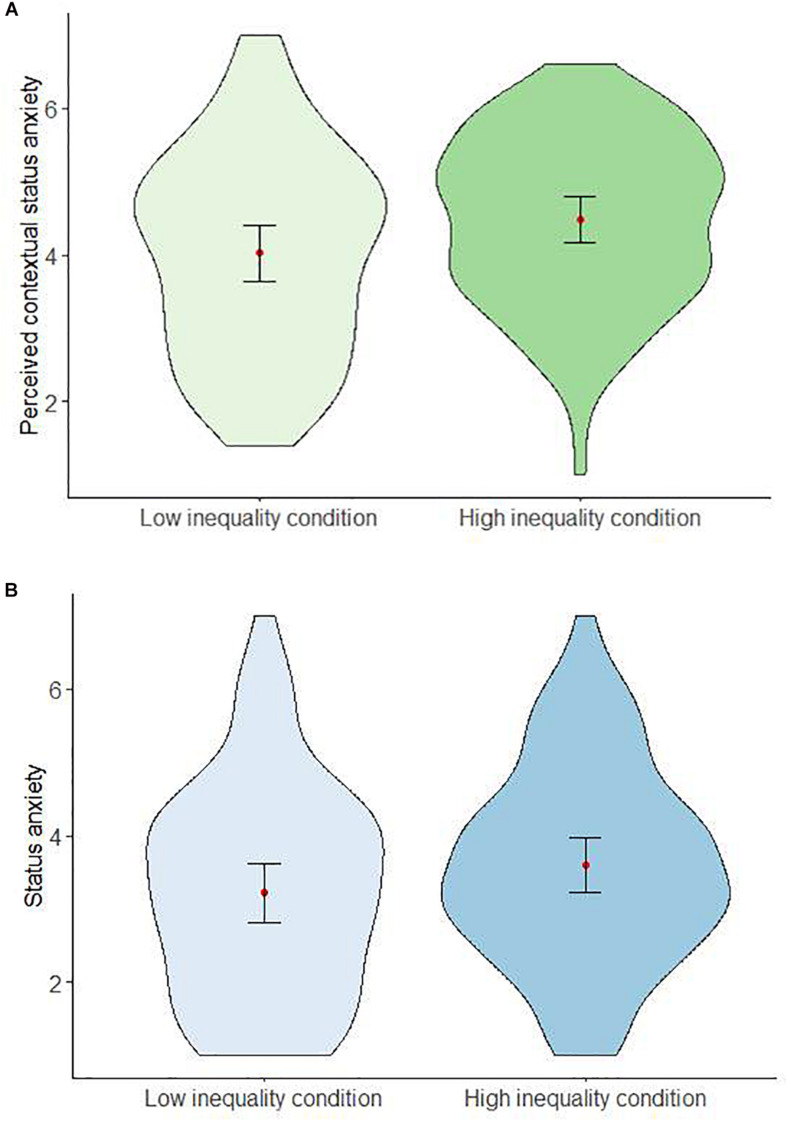
Effects of perceived economic inequality condition on perceived contextual status anxiety **(A)** and participants’ status anxiety **(B)** in study 2.

In support of Hypothesis 2, perceived contextual SA differed significantly between conditions (Figure 2A), *t*(183.08) = 2.53, *p* = 0.012; *d* = 0.36; 95%CI [0.08, 0.64]: participants in the low inequality condition perceived less SA among people in their income group (*M* = 4.03; SD = 1.39) than participants assigned to the high inequality condition (*M* = 4.49; SD = 1.15).

However, participants’ SA did not significantly differ between the high and low inequality condition (Figure 2B), *t*(198) = 1.88, *p* = 0.061; *d* = 0.26; 95%CI [−0.01, 0.55]. Thus, Hypothesis 1 was not corroborated.

#### Exploratory Analysis

**FIGURE 3:**
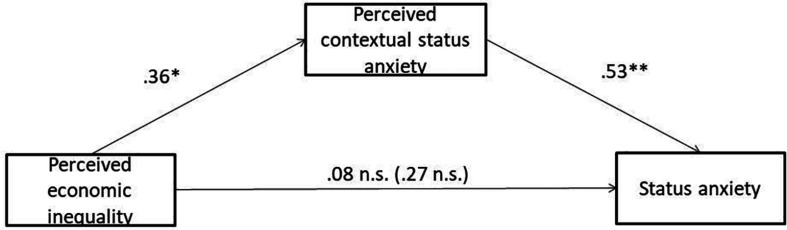
Indirect effect of perceived economic inequality condition on participants’ status anxiety through perceived contextual status anxiety in study 2. Coefficients are standardized; total effect in parenthesis; ^∗^*p* < 0.05; ^∗∗^*p* < 0.001.

We performed a bootstrap regression analysis (Hayes, 2017) to examine whether the inequality manipulation had an indirect effect on participants’ SA through perceived contextual SA. Indeed, we found that the inequality manipulation had an indirect effect on participants’ SA through the perceived SA of others in their income group (partially standardized indirect effect = 0.19; 95%CI [0.04, 0.35]; RMSEA = 0.141; CFI = 0.838; TLI = 0.787; SRMR = 0.085; Figure 3)^5^.

## Discussion

In Study 1, PEI uniquely predicted both SA and perceived competitiveness. Moreover, an indirect effect of PEI on SA was found, as PEI increased perceived competitiveness, which in turn increased personal SA. Results in Study 1 not only suggest that PEI increases SA, but also that perceiving the social environment as highly competitive could contribute to this effect. Thus, these results extend and bridge the gap between previous findings on the effect of economic inequality on the competitive normative climate on the one hand (Sommet et al., 2019; Sánchez-Rodríguez et al., 2019) and the SA theory on the other (Wilkinson and Pickett, 2017). However, the observational nature of this study does not allow the establishment of causal relationships. In order to accomplish this goal, PEI was experimentally manipulated in Study 2. Moreover, in Study 2 we investigated the effect of PEI on a more specific form of perceived competitiveness, based on the struggle for SES: namely perceived contextual SA.

In Study 2, results supported Hypothesis 2, as PEI was found to increase perceived contextual SA. On the other hand, results did not support Hypothesis 1, as PEI was not found to have a significant direct effect on participants’ SA. Exploratory analysis, however, revealed another indirect effect of PEI on SA, as perceiving similar others to be more concerned about their SES due to higher PEI gave rise to an SES-competitive mindset, further contributing to increase personal SA. These results suggest that perceived contextual SA may induce a competitive mindset that can favor status-oriented motives. On this matter, perceived SA could act as a descriptive norm, and as such, influence the motivation orientation (Manning, 2009). Importantly, PEI may indirectly affect participants own SA through this descriptive norm. However, this exploratory result should be treated with caution, given that it was not hypothesized.

At least three alternative hypotheses could explain the absence of a significant effect of PEI on participants’ SA. First, as this is the first time to our knowledge that the effect of PEI on SA has been experimentally tested, we did not have information about the effect size, in case of Hypothesis 1 being true. We could have therefore underestimated the required sample size to detect it. Second, it may not be socially desirable to admit one’s SA, so that participants could be censoring themselves. Third, although the experimental setup can manipulate the subjects’ judgment of low vs. high-inequality situations, it may be not sufficient to influence participants’ SA in the short term. After all, participants were asked to imagine their lives in a hypothetical society, and their feelings in this situation. For that matter, a more realistic context could better capture the contextual effect of economic inequality on SA. As these three alternative explanations have been created *post hoc*, further studies should test them.

On the other hand, although the exploratory results indicated that perceived contextual SA could lead to an indirect effect of PEI on SA, the study design does not allow the establishment of a causal relationship between the former and personal SA, as other alternative explanations could not be discarded (Spencer et al., 2005). For instance, it is possible that participants’ SA may influence perceived contextual SA, as participants may project their own feelings onto those of similar others. Further studies experimentally manipulating perceived contextual SA could help in supporting or disconfirming the indirect effects presented in this article.

Finally, the presented results may be taken with caution, as both studies were conducted in a relatively rich and moderately unequal country, and most of the sample came from working and middle class families. In modeling these effects in other contexts, country cultures as well as social class cultures have to be taken into account, especially regarding normative competitive climate and social standards of socioeconomic success. For instance, collectivistic and individualistic orientations can culturally vary between both countries and social classes (Markus and Kitayama, 2010; Kraus et al., 2012). Thus, future research should take these differences into account and explore their role in determining how income inequality affects personal or collective SA (i.e., concerns about in-group SES).

### Implications

To our knowledge, this is the first time that experimental evidence has been provided on the causal effect of PEI on perceived contextual SA, or on the indirect effects of PEI on personal SA. As the struggle for SES becomes more intense, the consequences for societies could be disturbing. Perceiving a generalized competition for a better position on the social ladder (i.e., perceived contextual SA), for instance, could dampen social cohesion and generalized trust, which have been found to predict healthy life expectancy, civic and political engagement, and general well-being (Uslaner and Brown, 2005; Elgar, 2010; Buttrick et al., 2017).

SA makes societies less cohesive and individuals more likely to engage in selfish and competitive behavior (e.g., conspicuous consumption; Walasek and Brown, 2015), and is related with poorer health and wellbeing (Paskov et al., 2013; Layte and Whelan, 2014; Buttrick et al., 2017). This research joins a growing body of evidence on the deleterious psychological effects of economic inequality (see Jetten and Peters, 2019), and on the urge to reduce it in all its forms (Wilkinson and Pickett, 2009).

Furthermore, the presented results could inform about both policies and interventions aimed at palliating the aforementioned effects. As PEI was found to increase personal SA only through perceived contextual SA, interventions should focus on the latter variable. Work settings that incentivize cooperation vs. competition, for instance, could dampen SA, contributing to higher job satisfaction and wellbeing (Keshabyan and Day, 2020). In the same vein, disseminating alternative normative messages oriented at reducing perceived competition based on material resources, and at increasing cooperation among low and middle classes for a general improvement in quality life, would contribute to building more cohesive and resilient societies. Messages promoting frugal behavior rather than materialism (Suárez et al., 2020), for instance, could defuel the SA normative climate, as well as political movements based on shared identities among low and middle classes.

## Conclusion

The present research provided evidence that economic inequality makes people feel that they live in a society where they are constantly concerned and competing with each other for their position based on the material resources they possess. Thus, reducing economic inequalities and working for less competitive and materialistic social environments would contribute to build healthier and more cohesive societies.

## Data Availability Statement

The datasets presented in this study can be found in online repositories. The names of the repository/repositories and accession number(s) can be found below: https://osf.io/9wevd/?view_only=65ed212f44804aa5a50fe1d933eef01a.

## Ethics Statement

The studies involving human participants were reviewed and approved by Comité de Ética en Investigación Humana (CEIH), Universidad de Granada. The patients/participants provided their written informed consent to participate in this study.

## Author Contributions

All authors conceived and designed the studies. DM carried out the studies, collected and analyzed the data, and wrote the manuscript with support of GW and RR-B. All authors discussed the results and contributed to the final manuscript.

## Conflict of Interest

The authors declare that the research was conducted in the absence of any commercial or financial relationships that could be construed as a potential conflict of interest.

## Footnotes

1 Other Hypotheses and analyses beyond the scope of this article were preregistered for Study 1. Preregistration, materials and data are available online (https://osf.io/h35uj/?view_only=c026d785644948ea945650cb88aa5ff3). Other variables were measured with exploratory purpose (see Supplementary Material).

2 Data retrieved from http://ec.europa.eu/eurostat/data/database

3 https://osf.io/d9tnh/?view_only=eabd42cbbc884c7b8b67b79e963ad2ca

4 https://osf.io/d9tnh/?view_only=eabd42cbbc884c7b8b67b79e963ad2ca

5 Exploratory analysis, as well as contrasts on manipulation check, SA and perceived contextual SA, was repeated controlling for participants’ sex, age, political orientation, SES and subjective SES. Results and conclusions did not differ from the ones presented here (see Supplementary Material).
