# Supplementary material for: Economic Inequality Increases Status Anxiety Through Perceived Contextual Competitiveness
Source: Front Psychol. 2021 May 24;12:637365. doi: 10.3389/fpsyg.2021.637365 (PMC8182636; doi:10.3389/fpsyg.2021.637365)
Supplement: Supplementary file 1 [file Data_Sheet_1.docx]

Supplementary Material

# Supplementary analyses in Study 1

## Measures

*Comparison tendency* was indicated by averaging comparison tendency in Spain and in the same reference groups to which perceived economic inequality was also asked for. Participants answered to what extent they agree with the following: ““I usually compare myself with people <in Spain / among reference group>”, from 1 (totally disagree) to 7 (totally agree). The resulting 5 items loaded on a single factor in EFA, explaining 62% of the variance (*α* = .84; *M* = 3.16, *SD =* 1.49).

## Supplementary exploratory analysis

The same least squares linear regression analyses on status anxiety as in Model 1 and Model 2 was performed. In Model 3, comparison tendency was added as predictor of status anxiety, in Model 4, the interaction term between subjective socioeconomic status and perceived economic inequality. While comparison tendency significantly and uniquely predicted status anxiety, the interaction term between subjective socioeconomic status and perceived economic inequality did not, that is, subjective socioeconomic status did not moderate the effect of perceived economic inequality on status anxiety. Model 3 and Model 4 are represented in Table S1.

Table S1

*Regression analyses’ results using Status Anxiety as the criterion*

|  | Model 3 | | Model 4 | |
| --- | --- | --- | --- | --- |
| Predictor | *b* | *b* 95% CI [LL, UL] | *b* | *b* 95% CI [LL, UL] |
| (Intercept) | 1.55 | [-0.01, 3.11] | 1.64 | [-1.03, 4.31] |
| Female | 0.28 | [-0.08, 0.63] | 0.28 | [-0.08, 0.63] |
| Age | 0.01 | [-0.01, 0.02] | 0.01 | [-0.01, 0.02] |
| Students^a^ | 0.21 | [-0.33, 0.76] | 0.21 | [-0.33, 0.76] |
| Unemployed^a^ | 0.36 | [-0.13, 0.84] | 0.35 | [-0.13, 0.84] |
| Political orientation | 0.08 | [-0.05, 0.20] | 0.08 | [-0.05, 0.20] |
| Education | -0.19** | [-0.32, -0.06] | -0.19** | [-0.32, -0.06] |
| Income decile | -0.03 | [-0.10, 0.03] | -0.03 | [-0.10, 0.03] |
| Subjective SES | 0.03 | [-0.11, 0.16] | 0.01 | [-0.43, 0.45] |
| Perceived economic inequality | 0.21* | [0.03, 0.39] | 0.19 | [-0.35, 0.72] |
| Perceived competitive climate | 0.25** | [0.07, 0.44] | 0.25** | [0.07, 0.44] |
| Comparison tendency | 0.28** | [0.15, 0.42] | 0.28** | [0.15, 0.42] |
| Subjective SES * Perceived economic inequality |  |  | 0.00 | [-0.09, 0.10] |
| Fit | *R^2^*  = 252**  95% CI[.14,.31] | | *R^2^*  = .252**  95% CI[.14,.30] | |
| Difference | Δ*R^2^* ^b^ = .045*  95% CI[.00, .09] | | Δ*R^2^*  = .000  95% CI[-.00, .00] | |

*Note.* A significant *b*-weight indicates the semi-partial correlation is also significant. *b* represents unstandardized regression weights.
* indicates p < .05. ** indicates p < .01.
^a^ contrasted against employed, self-employed or retired.

^b^ indicates Δ*R^2^* between Model 2 and Model 3.

As both perceived competitive climate and comparison tendency predicted status anxiety, a parallel mediation analysis was performed, to examine whether perceived economic inequality had indirect effects on status anxiety, simultaneously through perceived competitive climate and comparison tendency. Perceived competitive climate partially mediated the effect of PEI on SA (completely standardized indirect effect = .07; 95%CI [.01, .14]), as comparison tendency did (completely standardized indirect effect = .07; 95%CI [.03, .12]; RMSEA = 0.081; CFI = 0.758; TLI = 0.719; SRMR = 0.071; Figure S1)


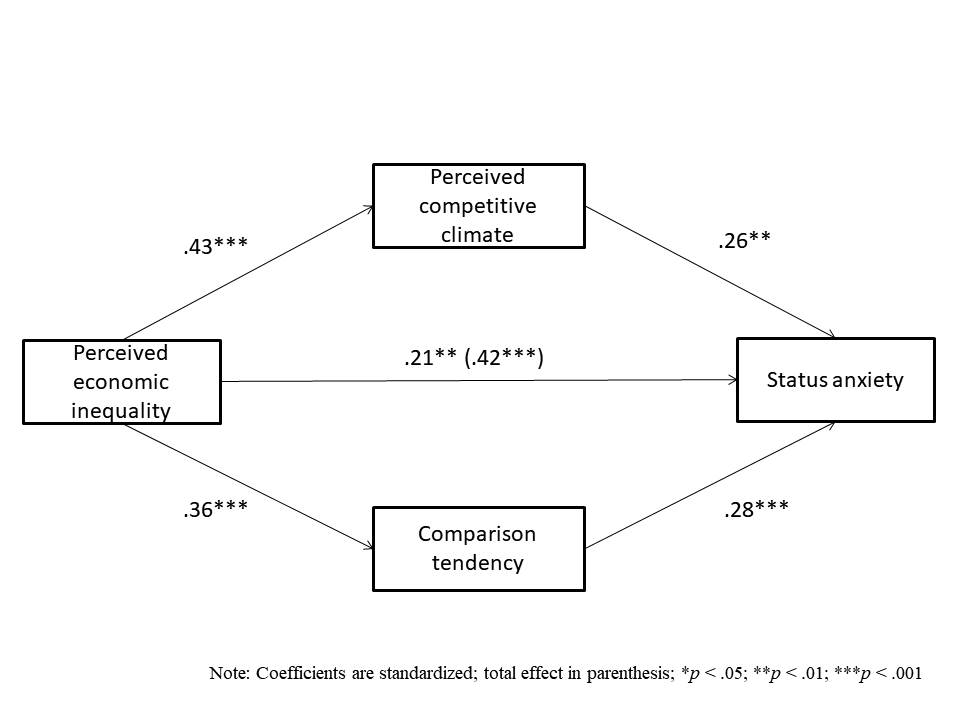


**Supplementary Figure 1.** Indirect effects of perceived economic inequality on status anxiety through perceived competitive climate and comparison tendency in Study 1.

# Supplementary analyses in Study 2

## Measures

*Perceived distance* from the lowest and the highest income group were measured by two items for each group (e.g. to what extent do you feel similar / different to the poorest /richest group?). Answers ranged from 1 (“not at all”) to 7 (“very much”). Items regarding the same income group were highly correlated (*r ≥* .65) so we averaged them.

*Perceived wealth* of the lowest and the highest income group were measured by one item for each group (i.e. how much wealth has group 1 /group 3 in Bimboola?). Answers ranged from 1 (“little wealth”) to 9 (“a lot of wealth”).

*Perceived lowest and the highest income groups’ status anxiety:* participants were instructed to rate their agreement with the items of two adapted version of Spanish version of the status anxiety scale (Melita et al., 2020), thinking about how other people belonging to group 1 /group 3 would feel in Bimboola (*α_poor group_* *=* .76; *α_rich group_* *=* .73).

*Subjective SES* (*M* = 5.65, *SD =* 1.51) and *political orientation* (*M* = 2.96, *SD =* 1.23) were measured as in Study 1.

## Supplementary exploratory analysis

### Main analyses with control variables

Three one-way ANOVAs were performed on manipulation check index, participants’ status anxiety, and perceived contextual status anxiety, with perceived economic inequality manipulation as fixed effect and sex, age, political orientation, SES and perceived SES as control variables. Perceived economic inequality manipulation was found to significantly increase perceived economic inequality (*F*(1,157) = 257.38, *p* < .001; *η_p_²* = .62), and perceived contextual status anxiety (*F*(1,157) = 5.86, *p* = .017; *η_p_²* = .04), but not participants’ status anxiety (*F*(1,157) = 2.78, *p* = .098; *η_p_²* = .02).

### Exploratory analyses with control variables

Two bootstrap regression analysis (Model 4 in Macro Process; Hayes, 2017) were performed on participants’ status anxiety, with perceived economic inequality manipulation as predictor, sex, age, political orientation, SES and perceived SES as covariates, and perceived contextual status anxiety or perceived ingroup wealth as mediators. Perceived economic inequality manipulation was found to have an indirect effect on participants’ status anxiety through perceived contextual status anxiety (standardized indirect effect = .19; 95%CI [.03, .37]), and perceived ingroup wealth (standardized indirect effect = .09; 95%CI [.01, .19]).

### Perceived wealth

A mixed model ANOVA was performed, with repeated measure in one factor (perceived wealth of poor, middle and rich group) ^[[1]](#footnote-1)^ and between participants’ comparisons in the other factor (low vs. high inequality condition). As can be observed in Figure S2, we found a within-participants main effect of income group (*F*(2,196) = 86.76, *p* < .001; *η_p_²* = .47)^[[2]](#footnote-2)^, indicating that participants perceived middle-income group (*M* = 5.81; *SD* = 1.10) being wealthier than low-income group(*M* = 3.49; *SD* = 2.60; *F*(1,197) = 174.34, *p* < .001; *η_p_²* = .47), and high-income group (*M* = 7.26; *SD* = 2.50) being wealthier than middle-income group (*F*(1,197) = 57.61, *p* < .001; *η_p_²* = .23). Significantly, although the total amount of income between all three income groups was the same in the high income inequality and in the low income inequality, we found an overall effect of inequality condition on perceived wealth (*F*(1,197) = 50.29, *p* < .001; *η_p_²* = .20) leading participants to perceive Bimbolean society as wealthier in the low inequality condition than in the high inequality condition. In particular, this effect was caused by differences in perceived wealth of the middle income group (participants’ in group) and low income group as indicated by the interaction between income group and inequality manipulation on perceived wealth (*F*(2,196) = 11.76, *p* < .001; *η_p_²* = .11) and subsequent post-hoc contrasts. While there was no significant difference between high and low inequality condition in high-income perceived wealth (*t*(176.89) = .82, *p* = .41; *d* = .12; *95%CI* [-.16, .39]), middle income group was perceived significantly poorer in high inequality condition than low inequality condition (*t*(197) = -3.53, *p* = .001; *d* = -.51; *95%CI* [-.79, -.22])) ,and so did low income group ((*t*(198) = -6.42, *p* < .001; *d* = -.91; *95%CI* [-1.20, -.62]).

**Supplementary Figure 2.** Perceived wealth of low, middle and high income in Study 2.

### Perceived status anxiety

A mixed model ANOVA was performed, with repeated measure in one factor (perceived status anxiety among poor, middle and rich group) and between participants’ comparisons in the other factor (low vs. high inequality condition). As can be observed in Figure S3, we found a within-participants main effect of income group (*F*(2,196) = 21.55, *p* < .001; *η_p_²* = .18)^[[3]](#footnote-3)^, indicating that participants perceived ingroup (*M* = 4.27; *SD* = 1.29) to feel more status anxiety than high-income group(*M* = 3.55; *SD* = 1.48; *F*(1,198) = 36.29, *p* < .001; *η_p_²* = .16). However, low-income group perceived status anxiety (*M* = 4.52; *SD* = 1.45) didn’t significantly differ from low-income ingroup’s (*F*(1,199) = 4.27, *p* = .040; *η_p_²* = .02). Also, we found an overall effect of inequality condition on perceived status anxiety (*F*(1,197) = 9.08, *p* = .003; *η_p_²* = .04). Although the interaction between income group and inequality manipulation on perceived status anxiety was not significant (*F*(2,196) = 2.83, *p* = .061; *η_p_²* = .03), the overall effect of inequality manipulation on perceived status anxiety was not the same across all income groups. In particular, while there was no significant difference between high and low inequality condition in high-income perceived status anxiety (*t*(197) = 0.12, *p* = .903; *d* = -.02; *95%CI* [-.30, .26]), perceived ingroup’s status anxiety was found to be significantly higher in high inequality condition than low inequality condition in the preregistered analysis, and so did low income group’s (*t*(198) = 3.39, *p* = .001; *d* = .48; *95%CI* [.20, .76]).

**Supplementary Figure 3.** Perceived status anxiety of low, middle and high income in Study 2.

# References

. Benjamin, D. J., Berger, J. O., Johannesson, M., Nosek, B. A., Wagenmakers, E. J., Berk, R., ... & Cesarini, D. (2018). Redefine statistical significance. *Nature Human Behaviour, 2*(1), 6-10. https://doi.org/10.1038/s41562-017-0189-z

1. Data on rich group perceived wealth from one participant was omitted in these analysis for being extreme outlier (differed more than 3 times the interquartile range from the median) [↑](#footnote-ref-1)
2. In all exploratory analyses alpha level of significance was set at *p* < .005, following recommendations from Benjamin et al. (2017). [↑](#footnote-ref-2)
3. In all exploratory analyses alpha level of significance was set at *p* < .005, following recommendations from Benjamin et al. (2017). [↑](#footnote-ref-3)
